# Tumor Growth Rate Determines the Timing of Optimal Chronomodulated Treatment Schedules

**DOI:** 10.1371/journal.pcbi.1000712

**Published:** 2010-03-19

**Authors:** Samuel Bernard, Branka Čajavec Bernard, Francis Lévi, Hanspeter Herzel

**Affiliations:** 1 Institute for Theoretical Biology, Humboldt University and Charité, Berlin, Germany; 2 University of Lyon, Lyon, France; 3 University Lyon 1, Faculty of Medicine Lyon Sud, Oullins, France; 4 INSERM, U776 “Rythmes biologiques et cancers”, Paul Brousse Hospital, Villejuif, France; 5 University Paris-Sud, UMR-S0776, Orsay, France; 6 Assistance Publique-hôpitaux de Paris, Chronotherapy Unit, Department of Cancerology, Paul Brousse Hospital, Villejuif, France; King's College London, United Kingdom

## Abstract

In host and cancer tissues, drug metabolism and susceptibility to drugs vary in a circadian (24 h) manner. In particular, the efficacy of a cell cycle specific (CCS) cytotoxic agent is affected by the daily modulation of cell cycle activity in the target tissues. Anti-cancer chronotherapy, in which treatments are administered at a particular time each day, aims at exploiting these biological rhythms to reduce toxicity and improve efficacy of the treatment. The circadian status, which is the timing of physiological and behavioral activity relative to daily environmental cues, largely determines the best timing of treatments. However, the influence of variations in tumor kinetics has not been considered in determining appropriate treatment schedules. We used a simple model for cell populations under chronomodulated treatment to identify which biological parameters are important for the successful design of a chronotherapy strategy. We show that the duration of the phase of the cell cycle targeted by the treatment and the cell proliferation rate are crucial in determining the best times to administer CCS drugs. Thus, optimal treatment times depend not only on the circadian status of the patient but also on the cell cycle kinetics of the tumor. Then, we developed a theoretical analysis of treatment outcome (TATO) to relate the circadian status and cell cycle kinetic parameters to the treatment outcomes. We show that the best and the worst CCS drug administration schedules are those with 24 h intervals, implying that 24 h chronomodulated treatments can be ineffective or even harmful if administered at wrong circadian times. We show that for certain tumors, administration times at intervals different from 24 h may reduce these risks without compromising overall efficacy.

## Author Summary

Chronotherapy of cancers aims at exploiting daily physiological rhythms to improve anti-cancer efficacy and tolerance to drugs by administering treatments at a specific time of the day. Recent clinical trials have shown that chronotherapy can be beneficial in improving quality of life and median life span in patients, but that it can also have negative effects if the timing is wrong. A theoretical basis for the rational development of individualized therapy schedules is still lacking. Here, we use a simple cell population model to show how biological rhythms and the cell cycle interact to modulate the response to cancer therapy. In particular, we show that the proliferation rate of cancer cells determines when treatments are most effective. We provide a simple formulation of the problem that can be used to compute an objective response function based on the drug sensitivity and the proliferation rate of tumor cells. Finally, we show that in some cases, treating at a different time every day may be more appropriate than standard daily chronotherapy. These results constitute an important step in designing individualized chronotherapy treatments, and point out to ways to design better clinical trials.

## Introduction

Neurons located in the suprachiasmatic nuclei (SCN) of the hypothalamus form a dominant circadian pacemaker that controls timing of many physiological processes, including cell cycle. The pacemaker integrates environmental cues and communicates timing information to peripheral organs, which respond appropriately to optimize their functions [1]. In host and cancer tissues, drug metabolism and susceptibility to the drug vary throughout the day. The characterization of daily rhythms in drug toxicity and efficacy was a foundation for the chronotherapy of cancer [2].

The main aim of anti-cancer chronomodulated treatment is to achieve an optimal balance between chronotolerance and chronoefficacy (drug tolerance and efficacy as a function of time of administration). However, because many circadian-dependent factors influence the outcome of a treatment, determining the optimal schedule has been difficult to implement in clinics [3]. Cytotoxic chemotherapy suppresses the hematopoietic system, and neutropenia is a major limitation to the doses of drug that can be tolerated. Therapeutic advantages of chronomodulated treatments are seen mainly in the tolerance to higher drug doses, along with a decreased severity of side-effects, rather than in the prolonged survival of the patients [4],[5].

The efficacy of a cytotoxic drug, at a given concentration, is given by the product between the fraction of cells sensitive to the drug and the fraction of sensitive cells killed by the drug. For cell cycle phase specific (CCS) drugs used in chronotherapy, the fraction of sensitive cells is defined by their cell cycle status (e.g. fraction of cells in S or M phase) [6]. The entry to S phase is induced by *c-MYC* and *cyclin D1*, and the entry to M phase is gated (blocked) by *WEE1* [7],[8]. Since those genes are controlled by the circadian clock, the cell cycle status is determined by the time of the day as well. Thus, drugs like cisplatin or 5-fluorouracil (5-FU) (S phase specific), docetaxel (M phase specific) and selicilib (G1 phase specific) would each be expected to have maximal efficacy and minimal toxicity at different times of the day.

Synchronization properties of the cell cycle to signals from the circadian pacemaker, namely phases and amplitudes, are tissue-specific. Blood cell progenitors [9], tongue epithelium [10], and cancer tissues [11] show tissue-specific daily variation in their DNA synthesis activity. In tumors, the response is perturbed and advanced-stage cancer cells can escape or even disrupt circadian control [12],[13]. Therefore, we would expect that the development of a cell cycle phase specific cancer chronotherapy strategy would depend on at least three circadian-dependent factors.

1. *The circadian time* of the patient, which defines the overall timing of physiological and behavioral activity relative to the daily environmental cues. There is a wide variation among individuals in the timing of their activity, and this is linked to the period length of the circadian pacemaker [14]. Isolated human fibroblasts display up to 4 h difference in the timing of the largest concentration of circadian proteins [15].
2. *The circadian status* of the host and the tumor, which defines how each cell type differs in its response to the circadian time of the patient. This is tissue-specific and defines the phases and amplitudes of the cellular activity in each tissue relative to the circadian pacemaker [16].
3. *The cycling status* of the host and tumor cells, which defines how cell cycle kinetic parameters differ between cell types, and how the circadian clock synchronizes the cell cycle. Because of variations in cell division times, this property is cell specific. Heterogeneity in tumor cell cycle kinetics also decreases the coherence of the circadian response. Together with the circadian status of the cells and the patient, the cycling status determines the daily peaks in DNA synthesis and cell division in the target tissues.

Here, we use a simple model of cell populations under circadian clock control and chronomodulated treatment to identify which biological parameters are important for the successful design of a chronotherapy strategy. We show that optimal CCS drug administration schedules, which minimize the sensitive fraction of the host cells and maximize the sensitive fraction of the tumor cells, are separated by 24 h intervals. However, if timing is wrong, a daily chronomodulated treatment schedule can lead to the worst therapeutic outcome as well. Using a theoretical analysis of treatment outcome (TATO), we show that clinically measurable cell cycle kinetics parameters are crucial in determining the response to CCS drugs. We show that chronomodulated treatments can be beneficial if tailored for individual patients, but can also be ineffective or even harmful if administered at wrong circadian times. We show that for fast growing tumors, administration times at intervals longer than 24 h may reduce these risks while maintaining a good overall efficacy.

## Results

### Numerical simulations of the behavior of the system with and without treatment

**Figure 1:**
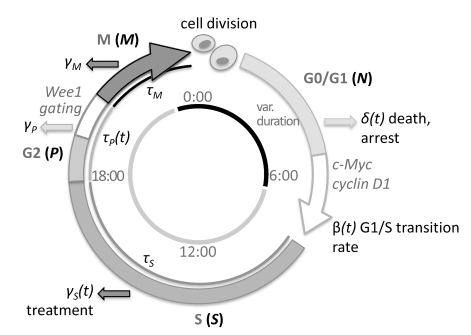
Cell cycle model. Cells progress along four phases: G0/G1, S, G2 and M. Transition from one phase to another depends on the circadian time. G1-S phase transition occurs at a rate 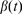. Cells in G1 phase can also leave permanently at a rate 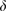. S/G2/M phases have fixed durations 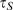, 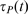, 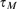. At the end of the M phase, cells divide and go back to the G0/G1 phase. Cells in S/G2/M phases die at rates 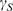, 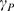, 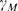. G1-S phase transition 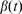 and G2 phase duration 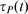 are clock-dependent (24 h periodic).

Renewing tissues have daily peaks in the fraction of cells in S phase [9]–[11]. To explore the influence of daily modulations of cell cycle kinetics on cell proliferation, we used a simple cell population model [17]–[19] (Figure 1). The cell population is divided into four phases: G0/G1, S, G2 and M. G1 phase has a variable duration controlled by the transition rate 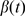 and S, G2 and M phases have a fixed duration 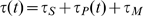. The circadian clock controls the G1-S phase transition and the G2 phase duration: the G1-S phase transition rate 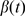 and the G2 phase duration 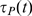 are 24 h periodic functions (see Methods for a more detailed description).

**Figure 2:**
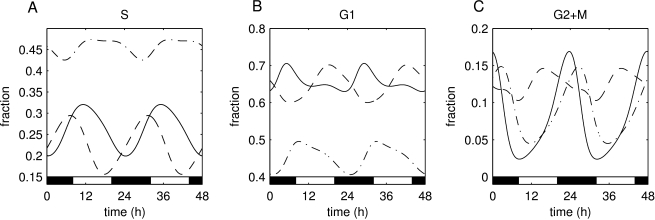
Daily evolution of the host, fast growing tumor and slow growing tumor. (A) S phase fraction. (B) G0/G1 phase fraction. (C) G2/M phase fraction. Dark phases are indicated by black bars (20:00 to 8:00). In panels A–C, time 0 corresponds to 72 h after beginning simulations, to allow for transients to vanish. Initial conditions (at 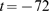 h) are 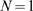, 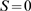, 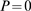, and 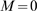. In panels A–C, solid lines denote host, dashed lines fast growing tumor, and dashed-dotted lines slow growing tumor.

We simulated time courses over 48 h for cell populations with different cell cycle phenotypes: host cells, tumor cells with a short S phase duration (fast growing tumors), and tumor cells with a long S phase duration (slow growing tumors). Because G1 phase has a variable duration (represented by an exponential distribution of times with parameter 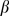), cells tend to desynchronize when there are no synchronization factors present. Even when cells are initially synchronized, once the clock control is off (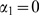), the fractions in each phase of the cell cycle reach a steady state within a few division cycles (asynchronous cell growth). While the clock control is on (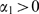), all populations, irrespective of their cell cycle length, show a circadian variation in the fraction of cells G1, S, G2 and M phases (Figure 2). The fraction of host cells in S phase varies from 20% to 30%, and peaks around 12:00 every day (Figure 2A, solid line). The fractions of tumor cells in S phase vary between 15% and 30% for fast growing tumors and between 42% and 47% for slow growing tumors, and they peak at different times (Figure 2A, dashed and dashed-dotted lines respectively). The fractions of cells in G1 and G2/M phases also peak at different times of the day and their amplitudes are different for each phase (Figure 2B, C). These results indicate that the fractions in each cell cycle phase match the circadian period but the time at which they peak is influenced by the cell cycle status (tumor and host cells respond with different strength to the external cues).

**Figure 3:**
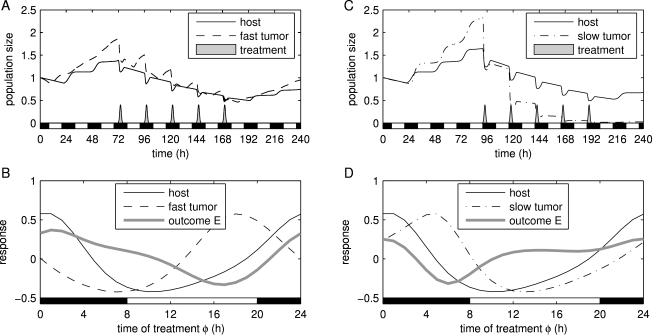
Treatment outcomes as a function of circadian time of administration. (A) Fast growing tumor treated at optimal time 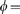
2:00. (B) Best treatment outcome for fast growing tumors (maximal 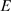) is at 2:00, while the worst is at 17:30 (thick line). (C) Slow growing tumor treated at optimal time 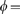
22:00. (D) Best treatment outcome for slow growing tumors is at 22:00, while the worst is at 5:30 (thick line).

S phase fractions in the host and tumor populations peak at different times, a feature that could be exploited by a well-timed administration of an S phase specific drug. We simulated the effect of one course of treatment based on a standard protocol (see Methods). We compared two tumor cell phenotypes: a fast growing tumor (Figure 3A,B) and a slow growing tumor (Figure 3C,D). Cell cycle kinetic parameters for the host and tumor cells were estimated from experimental data in patients when available; otherwise, data from mice were used. We assumed that the circadian clock acts at the same time of the day in the host and tumor cells, albeit more strongly on the host cells. To determine the optimal treatment time, we defined an outcome function 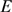 that measures the trade-off between anti-tumor efficacy and toxicity. We calculated the outcome of treatments given at different circadian times. The optimal treatment time for the fast and slow growing tumors is during night. However, the worst times of treatments are different: 17:30 for the fast growing tumor and 5:00 for the slow growing tumor (Figure 3B,D). This shows that the S phase duration alone can strongly affect the outcome of a chronomodulated treatment.

### Theoretical analysis of the treatment outcomes (TATO)

**Figure 4:**
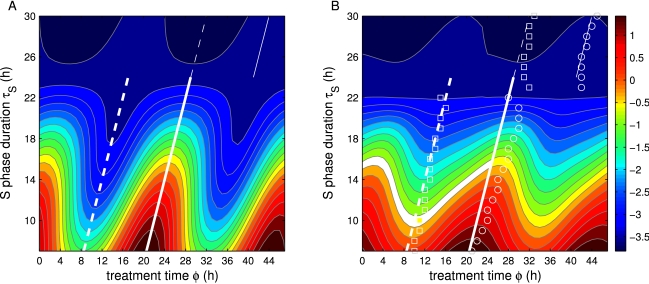
Response of host cells to treatment (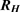) as a function of treatment time 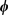 and S phase duration 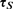. (A) TATO, as given by Eq. 1 with 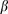 sinusoidal. (B) Numerical simulations of the full model. The response is normalized from low tolerance (blue) to high tolerance (red). Daily extrema predicted by TATO are indicated by white lines (dashed: highest toxicity 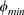, solid: lowest toxicity 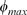, same in both panels). TATO predicts well the location of the extrema of the full model (squares: highest toxicity 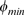; circles: lowest toxicity 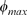). At 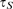 = 24 h, the location of extrema are shifted by 12 h (thick vs. thin lines).

The fraction of cells in each cell cycle phase determines how sensitive to treatment tissues are. Therefore, it would be useful to predict the best time of treatment based on kinetic data without having to run full simulations. We developed a theoretical method, TATO, to predict the influence of cell kinetics on CCS drug toxicity and efficacy. If the G1-S phase transition rate 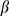 (due to circadian entrainment) and the surviving fraction 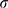 (due to the treatment) are 24 h-periodic, we can solve the periodic treatment problem by calculating the average host and tumor population growth rates under 24 h period perturbations. The contribution of the rhythmic entrainment of the cell cycle to the growth rate can be approximated by
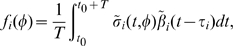
(1)where the subscript 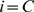 denotes the tumor and 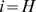, the host. (See Methods for a mathematical analysis). The value 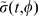 is the periodic component of the survival fraction of the cells that divide at time 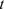, when treated at time 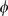. The value 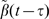 is the periodic component of the G1-S transition rate at time 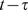. The integral, which is the average of the product between the two terms, is the net contribution of the periodic component to the rate of viable newborn cells over 24 h. As a function of 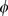, the sign of the integral determines the effect (positive or negative) of the clock and the treatment on the growth rate. We found that the integrals 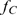 and 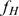 are good approximations of the response values 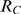 and 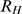 computed by numerical simulation (Figure 4).

The functions 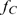 and 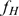, as approximations of response functions 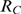 and 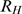, are useful to study the dependence of the treatment outcomes on the cell cycle kinetic parameters. For drugs targeting the S phase, three cell cycle parameters affect the periodic part of the growth rate: (1) the duration of the S phase 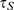, (2) the timing of the peak of the G1-S phase transition rate 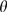, and (3) the timing of the cell death rate, given by the timing of the drug administration 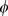. These parameters appear, explicitly or implicitly, in Eq. 1. The extrema of Eq. 1, which represent the largest and the smallest growth rates of the cell population, can be located when 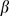 and 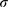 are known. As a first approximation, when the death rate 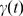 and the G1-S phase transition rate 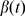 are sinusoidal and are largest at times 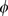 and 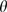, the location of the extrema can be calculated explicitly. The maximum of 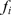 occurs when
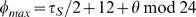
and the minimum of 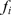 occurs when
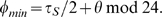
(Figure 4A, white lines). Therefore, to kill the largest fraction of cells, i.e. to minimize 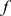, treatments should be applied halfway the S phase duration after the daily peak in G1-S phase transition. To spare the largest fraction of cells, the treatment should be applied 12 h later (detailed analysis in Methods). Based only on 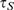 and 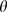, TATO predicts that the extrema of 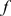 are 12 h apart. This approximation is good for 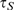 durations between 7 h and 24 h (Figure 4B). When 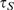 is larger than 24 h, the extrema are shifted by 12 h (Figure 4). When 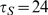 h, the timing of the treatment has no effect.

Anticancer drugs interfering with DNA synthesis (S phase) are widely used, but other phases of the cell cycle can be targeted as well. Therefore, in addition to the simulations for drug specific to S phase, we ran full model simulations for drugs acting on G1 or G2/M phase and compared the outcome to prediction from TATO (Table 1). The treatment protocol was the same as for the S phase drug, which is also included in Table 1. Optimal times of treatment in G1, S and G2/M phases vary by as much as 9 h between fast and slow growing tumors (formulas for optimal times are given in Methods). The worst times of treatment also show large differences between fast and slow growing tumors. Despite this, TATO predicts the optimal time within 2.5 h.

**Table 1:** Best and worst times of treatments.

phase	fast growing tumor				slow growing tumor			
	best		worst		best		worst	
	sim	TATO	sim	TATO	sim	TATO	sim	TATO
G1	23:00	1:00	7:00	13:00	14:00	16:15	5:00	4:15
S	2:00	4:30	17:30	16:30	22:00	19:45	5:30	7:45
G2/M	14:30	12:30	3:00	0:30	7:00	7:00	19:00*	19:00

The best and the worst times of treatments for cell cycle specific (CCS) anti-cancer drugs, based on treatment outcomes from numerical simulations (sim) and theoretical analysis (TATO). For S phase, the results compare to numerical simulations from Figure 3.

*: Range 13:00 to 1:00.

Taken together, these results indicate that TATO, using only a reduced set of kinetic parameters, can reliably predict the outcome of full simulations.

### Comparison of different chronomodulated designs

Previous computational studies have found that the fraction of cells killed with a constant drug infusion is higher (more toxic) than that killed with a chronomodulated infusion, for the same average killing rate [20]–[23]. Our model is consistent with these findings, and indicates that higher total doses of chronomodulated drug can be tolerated and are needed to achieve the same anti-tumor efficacy. These theoretical results are in agreement with clinical trials that showed consistent higher tolerance for chronomodulated compared to constant infusion [4], even when given at non-optimal times [24]. Lesser toxicity is independent from the circadian rhythms, i.e. chronomodulated treatments are less toxic even in absence of circadian rhythms 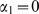. Thus, clinical and theoretical evidence shows that the shape of the infusion profile alone affects the treatment outcome significantly. For that reason, a direct comparison between constant and chronomodulated treatment is not really possible. Instead, we asked whether the same drug concentration profile administered at intervals different from 24 h could improve efficacy.

**Figure 5:**
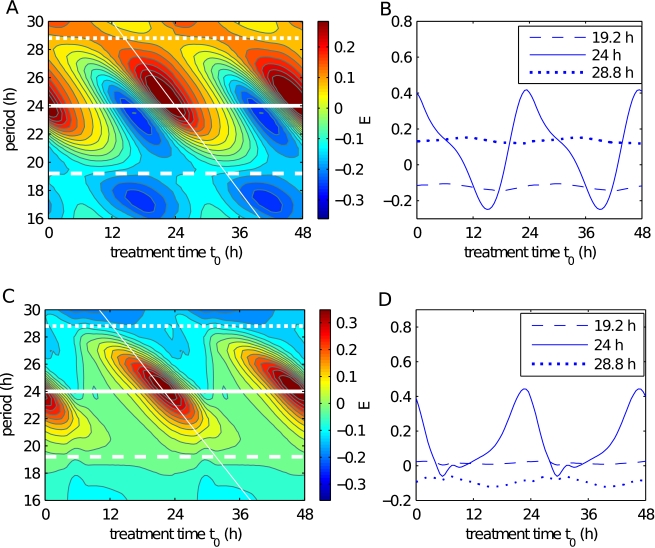
Treatment outcomes with different intervals between drug administrations. (A, C) Outcomes for fast (A) and slow (C) growing tumors, for different first day delivery times (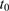) and intervals (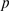) between administrations. The treatment outcome function used normalized, scaled responses 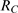 and 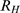 from simulations. Low values (blue) indicate bad treatment outcomes and high values (red), good ones. Outcomes for three intervals with 4.8 h difference (white lines) are compared: 19.2 h, 24 h, and 28.8 h. Eq. 2 predicts the location of the best response, as a function of 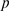 (thin white lines). (B, D) Outcomes at 24 h intervals show large amplitudes while small amplitudes occur at 19.2 h and 28.8 h intervals. For fast growing tumors, an interval of 28.8 h is a good alternative to 24 h (B, dotted line), but for slow growing tumors, an interval of 24 h is better (D, solid line).

We simulated the chronomodulated administration protocol with intervals ranging from 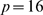 to 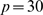 h, starting on the first day at a time 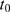 between 0:00 and 24:00. The total quantity and the infusion profile of the drug administered was the same for all intervals tested. Therefore, the resulting difference between outcomes depends only on the initial timing 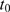 and the period 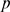. As expected, the largest amplitude of outcomes as a function of 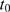, and the best outcomes globally, are at intervals 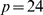 h (Figure 5A–D, solid lines). Likewise, the worst treatment outcomes also occur at intervals of 24 h.

To avoid the worst outcomes, it may be safer to seek treatment intervals that minimize outcome amplitudes, while optimizing the average outcome (maximizing 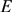). When 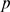 is close to 24 h, the treatment times can be averaged over the treatment course and TATO predicts an outcome given by
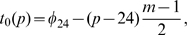
(2)where 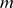 is the number of drug administrations during one course of treatment, and 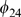 is the phase of a 24 h interval treatment. If 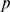 is larger than 24 h, the starting time of treatment 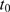 needs to be advanced to produce an outcome equivalent to the one obtained at 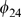. Here, using 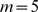, each hour increment in 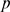 leads to a 2 h-advance in the starting treatment time. When 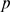 is much different from 24 h, i.e. 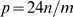, 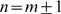, the average treatment phase is undefined, and TATO predicts an outcome independent from 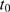. In both fast and slow growing tumors, at these values 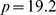 h and 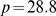 h, the outcome 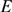 depends little on 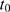. These two intervals offer circadian-independent treatment controls for the chronomodulated treatment (Figure 5B,D dashed and dotted lines). For a 24 h interval treatment to be safe to use, the time window during which the treatment is better than control should be large. TATO predicts that the outcome at 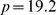 h and 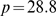 h depends significantly on the duration of the sensitive phase (Eq. 24 in Methods). Treatment intervals longer than 24 h are predicted to spare the most host and slow growing tumor cells while shorter intervals are expected to spare the most fast tumor cells. Numerical simulations confirmed that the outcomes depend on the intervals in a way that is specific to the tumor. Fast growing tumors showed the best response at intervals 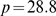 h except for a small time window around midnight (Figure 5B), while the slow growing tumors showed a better response at 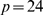 h (Figure 5D).

Differences in the cell cycle lengths between the tumor and host cells could be exploited by adapting the interval between drug administrations [25],[26]. Cell cycle length effects were also observed in the model in the presence of the circadian clock. Overall, a long interval tended to improve anti-tumor efficacy in fast growing tumors, while a short interval was detrimental (Figure 5A). The opposite was observed for slow growing tumors, where shorter treatment intervals had a better outcome (Figure 5C). This indicates that the cell cycle kinetics interacts with the timing of the drug administration to modulate outcomes, even in the presence of a circadian clock.

## Discussion

Several randomized clinical trials have demonstrated significant improvements in tolerability and antitumor efficacy of chemotherapy with standardized chronomodulated administrations in comparison with a constant rate infusion of chemotherapy [27] or a chronomodulated delivery with an opposite timing [28],[29]. However, these studies did not show any survival benefit. In a recent large trial involving colorectal cancer patients, standardized chronotherapy achieved significantly better survival as compared to conventional treatment in men, but not in women [30]. This indicates that the response of patients to standardized chronotherapy can be heterogeneous, and that there is a need for tailoring delivery pattern to an individual patient or to subgroups of patients with distinct chronotherapeutic determinants.

These determinants are structured in different levels: whole body/systemic, target tissues, and cellular levels. A combination of these three factors contributes to the therapeutic advantage of chronomodulated delivery in an individual patient, and to the best delivery time. Systemic level includes the main behavioral and physiological characteristics like sleep/wake and eating patterns. The phase difference in peak expression of clock genes of each chronotype indicates that the optimal treatment time could vary at least by 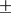
2 h [15]. For example, the efficacy and toxicity of 5-FU are dependent on thymidylate synthase (TS) activity, its molecular target [31],[32], and dihydropyrimidine dehydrogenase activity (DPD), the enzyme responsible for the elimination of 5-FU [33]. Circadian rhythms in both TS and DPD activity have been detected [34],[35]. TS activity is higher during S, G2 and M phases, therefore the rhythms might be due to cell cycle synchronization [36],[37], or to direct circadian clock control. Also, circadian rhythms in DPD activity modulate 5-FU concentration during the day, regardless of whether 5-FU delivery is constant or chronomodulated.

In this study, we showed how cell cycle kinetics, i.e. cell cycle length and duration of the susceptible phase, can affect the timing of the optimal chronomodulated treatment. We used a mathematical model for normal cell and tumor growth under circadian regulation to investigate: (i) how we can use differences of cell cycle dynamics between host and tumor cells to establish an optimal treatment schedule, and (ii) how timing of the best and the worst treatment outcomes depends on individual chronotype and the growth rate of the tumor.

Optimization of treatment schedules based on cell cycle kinetics of target tissues has been explored before [26],[38]. These experimental and theoretical studies were based on the concept of resonance therapy, where treating at integer multiples of the cell cycle length leads to a reduction of killing of normal cells. This could be exploited in cancers where tumors cells have a cell cycle time distinct from normal cells, or where there is a large variability in tumor cell cycle times. It was noted, however, that heterogeneity in normal cell cycle times reduces the benefits of resonance therapy [25]. These alternative schedules have so far received little attention in the context of chronotherapy.

Recently, Altinok et al. [39] used a computational approach based on cellular automata to explore the effect of the variability in the cell cycle length on chronotolerance and chronoefficacy of 5-FU and oxaliplatin. Their model accounted for the observation that the toxicity profiles of 5-FU and oxaliplatin are antiphase, and showed how variability in cell cycle lengths reduces the benefits of chronomodulated treatments. Cell populations with cell cycle times just below 24 h are most likely to benefit from chronotherapy, a result that could be explained by a synergy between cell cycle times, circadian rhythms and periodic treatments.

### Importance of the tumor growth rate

We have developed an analytic method, TATO, that allows us to identify the optimal treatment time based on the circadian status and on the cell cycle kinetics of the host and tumor tissues. TATO measures the average differential growth rate of host and tumor cells that is caused by the circadian modulation of the cell cycle. Three parameters are essential to calculate the differential growth rate: the G1/S phase transition rate, the duration of the drug susceptibility phase, and the death rate. Our model indicates that the cell cycle length, which can vary from 18 h to over 100 h in colorectal cancers [40], is important to determine the best treatment times and intervals.

24 h interval treatments at the right time provided the best efficacy. Yet, the worse time of treatment can be as near as few hours from the optimal time [41], making it risky to treat at 24 h intervals. A previous study has found a significant correlation between S phase duration and 5-FU sensitivity [36]. Here we showed that for fast growing tumor (short S phase duration), administering a drug that targets the S phase of the cell cycle at 28.8 h intervals may be safer than treating at 24 h intervals. However, we found that for slow growing tumor (long S phase duration), treating at 24 h intervals was indeed the best option, even when deviating from the optimal time. So far, schedules different from 24 h have not been tested in the context of circadian chronotherapy, but in this paper, we show that for fast growing tumors they might be a safer strategy.

### Quantitative approach to chronotherapy in a clinical setting

Drugs and the active drug metabolites used in chronotherapy are rapidly eliminated after delivery, which causes large modulations in their concentrations during the day. For that reason, patients with decreased 5-FU clearance rate due to a partial or complete loss of DPD activity might not benefit from chronomodulated treatments. An observed lower mean and amplitude of DPD activity in women is a possible explanation for the lower survival time with chronotherapy [5].

Here, we suggest how to individualize chronomodulated treatment schedules. First, patients with no overt circadian rhythm perturbations need to be selected, and their tumor kinetics assesed by measuring the S phase duration (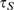) and potential doubling time (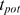). If the S phase duration of the tumor cells is short, a non-24 h schedule may be preferable. If the S phase duration of the tumor cells is long, a 24 h schedule could be more effective.

Second, the best treatment time could be determined using TATO. Constant infusion is not the best control for 24 h schedules since the shape of the infusion profile is likely to have a significant effect on outcomes [3]. Chronomodulated treatments with intervals spanning the whole day equally allows minimizing circadian effects, thus they could make suitable controls. Unlike for 24 h schedules, a constant infusion control group could be used to assess the efficacy of non 24 h interval treatments.

Third, once the optimal treatment time is determined, reverse pharmacokinetics could be used to retrieve the corresponding dose delivery schedule. Given a fixed dose 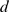 delivered to a tissue at time 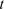, the fraction of surviving cells depends on the fraction of sensitive cells and the killing rate. If the killing rate varies in a predictable way during the day due to metabolism or elimination, it is possible to find a normalization dosage profile 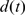 to make the killing rate time-independent. Thus, by knowing the quantity of drug needed to achieve a given killing rate, the fraction of surviving cells can be determined by the fraction of sensitive cells given by the model presented here.

The accepted administration time for 5-FU, 4:00, is based on the observation that in mice, the maximal tolerance is reached 5 h after light onset, corresponding to 5 h after beginning sleeping at 23:00 in humans [4]. In a recent study [28], 8 groups of patients received chronomodulated 5-FU-LV with peak times staggered every 3 h. Toxicity showed a marked circadian dependency of timing of chronomodulated 5-FU with leucovorin and oxaliplatin or carboplatin in cancer patients, with optimal time of 5-FU in cancer patients near 4:00 with 90% confidence limits. This study also showed more toxicity and large variability in women. Chronomodulated drug infusion differs in two respects from constant rate infusion: modulated concentration profile and timing. Chronotherapy is based on adapting the timing of treatment regimens to the circadian rhythms [27]. Thus, for the chronotherapy principle to work once the effect of concentration profile is discounted, there should be a 12 h time window during which the therapeutic outcome improves. This means that only 6 h would separate the optimal treatment time and a no-effect treatment time. We conclude that for chronotherapy clinical trials, patients need to be grouped according to the chronotype, tumor growth kinetics and pharmacokinetics/pharmacodynamics characteristics.

## Methods

### Population model of cell proliferation with circadian control

The cell population is divided into four phases: G0/G1, S, G2 and M. The G0/G1 phase includes cells that are actively dividing, but are in the pre-DNA synthesis or growth phase (G1) and cells that are quiescent but can be recruited to the cell cycle (G0). The S phase includes cells in DNA synthesis. The G2 and M phases include cells that have synthesized DNA and are progressing through mitosis. We used a population model of cell proliferation [17]–[19] in which we introduced a circadian control (Figure 1). Each stage of the cell cycle and its relationship to the circadian clock is modeled. The input to the model is a treatment course and the output is the population size in each cell cycle phase at any given time of the day.

We consider two cell types, host and tumor cells. Cell kinetic parameters for the host correspond to blood cell progenitors and for the tumor, to colorectal cancer cells. The model tracks the total cell number and fraction of cells in each phase for host and tumor during a course of chemotherapy, allowing estimates of efficacy and toxicity. The equations for the cell populations are
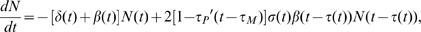
(3)

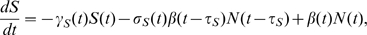
(4)

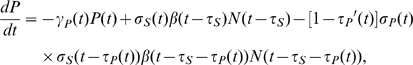
(5)

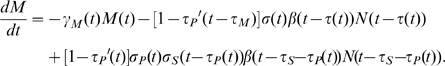
(6)


Each equation represents the balance between fluxes of cells (cells/hours) entering (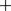 terms) and leaving (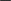 terms) a cell cycle phase (see Figure 1 for details about the model). 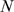 (Eq. 3) is the G0/G1 phase cell number, 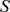 (Eq. 4) the S phase cell number, 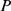 (Eq. 5) the G2 phase cell number, and 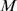 (Eq. 6) is the M phase cell number. The total cell number is denoted 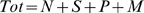.

The term 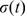, 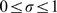, is the fraction of cells surviving the cell cycle (S/G2/M phases) at time 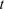. It is the product of phase specific survival rates,
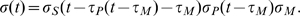
(7)Time delays (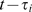) account for the finite time required for cells to progress through each phase. The survival rates for the S, G2 and M phases are determined by integrating the phase-specific death rates 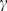 over the duration of each phase,
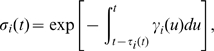
(8)where 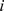 is one of 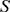, 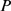, 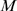. The duration 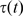 is the total length of S, G2, and M phases of cell dividing at time 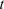,
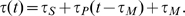
(9)


The phase and amplitude of 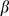 are given by 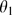 and 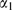. Similarly, the phase and amplitude of 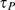 are given by 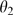 and 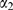 (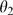 and 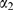 are relative to 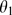 and 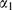). A sinusoidal circadian input with a specific phase and amplitude is assumed for 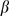 and 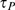,
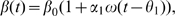
(10)

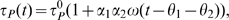
(11)where the circadian function is
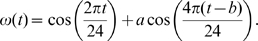
(12)The coefficient 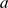 and phase-shift 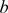 are set for all simulations to 0.2 and 14 h respectively. The function 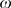 mimics the typical expression profile of circadian genes in many tissues, for a given individual. Note that circadian rhythm variability among individuals affect these parameters.

Kinetic parameters for bone marrow (host) and colorectal cancer (tumor) are derived from experimental data or were adjusted using this model. For the bone marrow 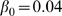 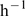 [25], 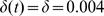 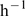 [25], 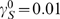 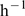 [25],[40], 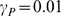 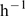, 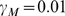 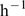, 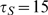 h [25], 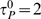 h, 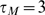 h, 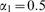 [9], 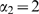 [8], 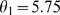 h [9], 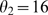 h [8]. For the tumors, parameters are identical except 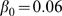, 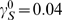 (fast), 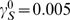 (slow), 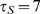 (fast), 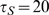 (slow), 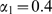.

The population model is linear and simulations of host and tumor cell growth show that their cell numbers grow exponentially with a circadian modulation. Here we neglect nonlinear terms that would eventually cause the cell number to stabilize. We assume that with the treatment, the cell number is far from equilibrium. For a small-size tumor, this is a reasonable assumption. We also neglect the systemic feedback mechanisms of normal tissue homeostasis, which are more relevant to study between courses of chemotherapy when patients are recovering. Therefore, a linear model is also considered for the host tissues under cytotoxic stress.

### Simulation of different treatment schedules

We simulate a colorectal cancer treatment with 5-FU [42],[43]. 5-FU is an S phase specific drug that inhibits thymidylate synthase activity required for DNA synthesis, and consequently induces cell death. Chemotherapy schedules used clinically are either chronomodulated at 24 h intervals, or a constant infusion of 5-FU for a few consecutive days. The treatment is repeated every two to three weeks [4].

For simplicity, we simulate only one course of chemotherapy. We consider three different schedules: chronomodulated with 24 h intervals, flat infusion, and chronomodulated with intervals different from 24 h. One course of treatment lasts 5 days or 5 chronomodulated administrations. To isolate the effect of chronomodulation of treatment, we ignore the pharmacodynamics/pharmacokinetics aspects and we assume that chemotherapy acts on tumor and host cells in the same way. Because cytotoxic chemotherapy affects the hematopoietic system, and neutropenia is a major limitation to drug tolerance, we simulate the effect of 5-FU with blood cells as the host tissue.

The effect of 5-FU is simulated by adding a drug-induced death rate to the basal apoptosis rate of S-phase cells,
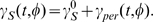
(13)The chronomodulated drug-induced death rate, 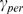, takes the form of a truncated Gaussian function centered at circadian time 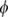, the treatment time (between 0 and 24 h),
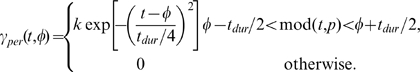
(14)Drug administration is repeated at intervals of 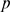 hours. The duration of drug infusion is 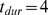 h [4]. The coefficient 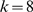 is the maximal drug-induced cell death rate. The equivalent flat rate infusion (normalized so that it kills the same fraction of cells than the chronomodulated infusion, in one day) is the constant
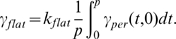
(15)The normalization factor is 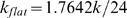.

For all simulations, the initial conditions were set to 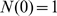, 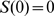, 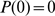, 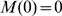 and 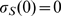 (total number initialized to 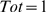). With the parameters chosen, the relativepopulation is quickly synchronized by the circadian rhythm.

Numerical simulations were performed with the Volterra solver of the package XPPAUT. Analysis was done with Matlab 7.0. Codes (XPPAUT and Matlab) are available as supplementary text (Texts S1, S2, S3,S4).

### Measure of treatment outcomes

The treatment outcome measure is defined as
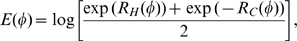
(16)where the functions 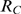 and 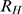 measure the cytotoxicity in tumor (C) and host (H) cells. The parameter 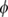 is the circadian time of drug administration in case of a 24 h treatment interval. For non-24 h intervals, it is the time of administration on the first day of treatment. 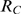 and 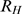, obtained from numerical simulations, are the normalized cell numbers 7 days after the first day of treatment 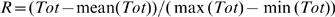, where 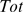 is the total cell number as a function of 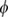. The outcome function *E* must increase with 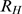 (high tolerance) and decreases with 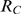 (high killing rate). For the flat infusions, *E* is constant. Close to zero, a Taylor expansion gives
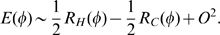
(17)The outcome 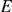 measures the difference between responses 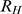 and 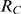, and penalizes both excessive toxicity and poor anti-tumor efficacy. An optimal treatment maximizing tumor cell kill and minimizing host cell loss is found by maximizing the outcome function 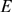.

### Theoretical analysis of treatment outcomes (TATO)

Equation 3 does not depend on other dynamical variables, so its stability analysis is simplified. Assuming a exponential growth, 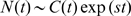, where 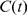 is a 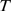 = 24 h-periodic function and 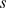 is the growth rate, we have from Eq. 3,
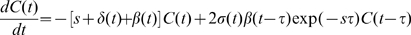
(18)Taking the average over a period, we obtain
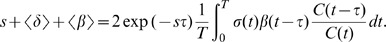
(19)For cell death occurring in the S, G2 or M phase, the death rate 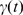 is chronomodulated. By making the simplifying assumption that the function 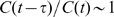, 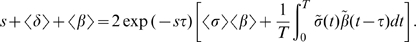
(20)The angle brackets denote the average over a period and the tildes the remaining, oscillatory part with a zero average. Thus, periodic parameters act only on 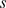 through the integral term,
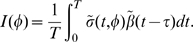
(21)The integral can be either positive or negative, modulating the growth rate accordingly. As a consequence, the growth rate (tolerance) is maximal when the integral is maximal and the death rate (toxicity) maximal when the integral is minimal.

We consider 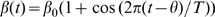 and a drug specific to the S phase 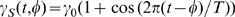. Then,

1. The integral is maximal at 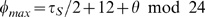.
2. The integral is minimal at 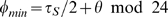.
3. The amplitude of the integral, 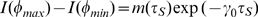, where the function 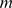 is a symmetric function on 0–24 h, 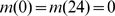 and the value 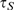 that maximizes 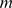 is 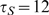 h.

The values 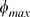 and 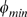 are shifted 12 h when 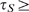
24 h. If the drug acts on the G2/M phases, with 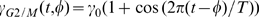 then

1. The integral is maximal at 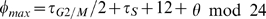.
2. The integral is minimal at 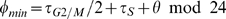.

For cell death occurring in the G1 phase, the death rate 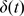 is chronomodulated. We assume that 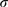 is constant and therefore, the integral term becomes
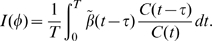
(22)If 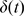 peaks at 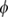, meaning many cells in G1 are lost, the periodic solution 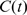 will reach a minimum value at 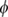. Thus the ratio 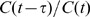 will have a maximum at 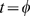 and a minimum at 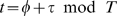. Assuming that 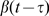 peaks at 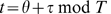 and is minimum at 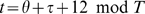,

1. The integral is maximal between 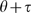 and 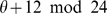.
2. The integral is minimal between 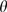 and 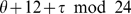.

When treatment intervals are different from 24 h, the outcome will depend on the administration times over the whole course of treatment. If 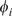 is the time of the 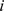-th administration, the effect on the growth rate is
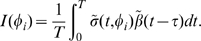
The average effect of 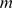 successive administrations at times 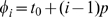, 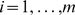 is
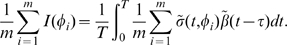
(23)When 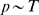, it is justified to replace the term 
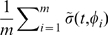
 with 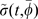, where
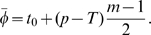
Therefore, the outcomes will be equivalent when 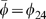, with 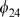 the phase of the 24 h interval treatment. The starting treatment time must then be
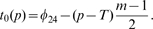
(24)When 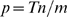, with 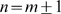, administration times are distributed equally around the circadian period and 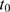 has little effect on the outcome. Neglecting the circadian clock allows computing the treatment intervals that minimize the growth rate of the equation 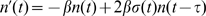, with a 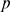-periodic survival fraction 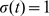 if 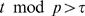 and 0 otherwise. This means that all cells in the sensitive phase are killed at intervals 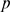. The minimal growth rates occurs at values 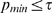, since not a single cell would come out of the sensitive phase alive. The maximal growth rate occurs when 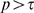 and the fraction of cells in the sensitive phase is minimal. Let 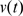 be the cell number in sensitive phase, given by 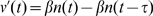. Right after administration, 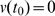. The sensitive fraction reaches a minimum when 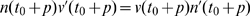. This occurs a time 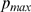 after the last administration, where
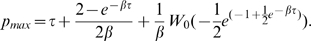
(25)

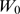
 is the Lambert W function, and satisfies 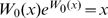.

## Supporting Information

Text S1 XPPAUT file to simulate population growth and treatment.
(0.01 MB TXT)

Text S2 Matlab file to run the model file cccMatlab.ode.
(0.01 MB TXT)

Text S3 Matlab script to load data obtained from model simulations.
(0.01 MB TXT)

Text S4 Script to extract data from data sets with two indices.
(0.01 MB TXT)
